# Influence of Altered Sleep Patterns on Health-Related Quality of Life and Academic Performance Among Dental Students: A Cross-Sectional Study

**DOI:** 10.7759/cureus.86624

**Published:** 2025-06-23

**Authors:** Agna Sabu, Parvathy Ghosh, Sarika K, Sangeeth KS, Sapna Varma NK, Ajith Vallikat Velath

**Affiliations:** 1 Orthodontics and Dentofacial Orthopedics, Amrita School of Dentistry, Amrita Institute of Medical Sciences, Kochi, IND

**Keywords:** academic performance, dentistry, excessive daytime sleepiness, quality of life, sleep quality

## Abstract

Background

Sleep plays a vital role in maintaining cognitive function, emotional well-being, and academic performance. Dental students often face unique stressors, such as demanding academic schedules, clinical responsibilities, and irregular sleep patterns, which may negatively affect their sleep quality and overall health. In the Indian context, limited research has explored how sleep-related issues impact both the academic and clinical performance of dental students. This study aimed to assess sleep quality, daytime sleepiness, sleep deprivation, and health-related quality of life (HRQoL), and to examine their association with academic performance among students of a dental college in India.

Materials and methods

A cross-sectional study was conducted among 102 undergraduate dental students of Kerala. Validated questionnaires were distributed through electronic mail using Google Forms (Google, Inc., Mountain View, CA, USA), which assessed sleep quality using the Pittsburgh Sleep Quality Index (PSQI), daytime sleepiness using the Epworth Sleepiness Scale (ESS), HRQoL using the SF-36 questionnaire, the Sleep Deprivation Index (SDI), and academic performance using self-reported grade point average (GPA). Pearson correlation tested associations, with p ≤ 0.05 considered significant.

Results

Of the 102 students, 83 were females and 19 were males, with a mean age of 20 years. The mean ESS score was 8.40 ± 4.66, and 30.3% (n = 31) of students exhibited daytime somnolence. The mean PSQI score was 6.29 ± 2.5, and 63.7% (n = 65) of students had a global score greater than 5, indicating poor sleep quality. HRQoL was evaluated under eight different categories, with the domains of fatigue, emotional well-being, and emotional problems showing lower scores. The relationship between quality of sleep and academic performance revealed a strong negative correlation (Pearson correlation = -0.796, R² = 0.634, p = 0.00), which was statistically significant. However, there was no statistically significant association between excessive daytime sleepiness and academic performance.

Conclusions

The present study concluded that poor sleep quality, as determined by a higher PSQI score, was associated with poor academic performance. Additionally, the dental students frequently experienced excessive daytime sleepiness and psychological distress.

## Introduction

Sleep is essential for humans to perform many essential physiological functions, and the quantity and quality of sleep needed are influenced by a variety of interrelated environmental conditions and physiological processes. Sleep deprivation often leads to emotional instability, high levels of stress and anxiety, judgmental errors, and lower levels of performance [1].

The prevalence of sleep disturbances in the general population is high, and medical/dental students are highly susceptible to poor sleeping patterns [2]. The high prevalence of sleep issues among dental students is caused by several factors, including long study and class hours, preclinical and clinical work, mental stress, unhealthy lifestyle habits, and extensive use of virtual social media [2]. 

Evidence suggests that getting enough restful sleep is essential for maintaining both physical and mental well-being, as well as for long-term learning, neurocognitive function, and psychomotor performance [2-4]. In addition, insufficient sleep might heighten an individual's vulnerability to anxiety and depressive disorders [5]. Academic performance is influenced by four essential sleep characteristics: quantity, quality, regularity, and sleep phase timing. Previous studies across different populations and academic levels have found that short sleep duration, poor quality of sleep, late bedtimes and rise times, and irregular sleep cycles had a significant negative impact on students' academic performance [2,6]. The majority of these studies concluded that students who had sleep disturbances or poor sleep quality were more likely to have psychological problems and low academic performance. Academic achievement also depends on other non-academic factors, such as the management of psychological distress. Indeed, much research has indicated that elevated stress levels may impact an individual's concentration and academic performance.

To the best of our knowledge, there is no previous literature available evaluating altered sleep patterns, health-related quality of life (HRQoL), and academic performance among dental students in Kerala, India. Thus, the aim of this study was to assess sleep quality, daytime sleepiness, and sleep deprivation, and to examine their association with HRQoL and academic performance among undergraduate dental students in Kerala, India.

## Materials and methods

This cross-sectional study was conducted among undergraduate dental students of Amrita Vishwa Vidyapeetham University, Kochi, India, from the first year to the final year. The sample size was calculated based on the results for the mean and standard deviation (SD) of sleep quality using the Pittsburgh Sleep Quality Index (PSQI) questionnaire, and the sleepiness score using the Epworth Sleepiness Scale (ESS), as reported in a previous study by Caldeira et al. [7]. With 10% relative precision and 95% confidence, the minimum sample size was 72.

All students were invited to participate by electronic mail, and their participation was voluntary and anonymous. Over a period of two months, 102 students responded. Questionnaires were distributed to consenting students via electronic mail, using an online data collection tool called Google Forms (Google, Inc., Mountain View, CA, USA). The Institutional Ethical Board at Amrita Institute of Medical Sciences approved the study protocol (Ethical Number: ECASM-AIMS-2023-427).

Validated questionnaires were used to measure quality of life, sleep quality, and sleepiness index. The PSQI questionnaire was used to assess sleep quality [6], the ESS assessed daytime sleepiness [8], and the SF-36 questionnaire [9] assessed HRQoL under different conditions. PSQI is a self-rated questionnaire that has 19 items to evaluate subjective sleep quality. The questionnaire yields a general score ranging from 0 to 21 and measures seven aspects of sleep quality; poor sleep quality is indicated by higher scores. Values over 5 are seen as indicative of poor sleep quality. The ESS questionnaire has eight self-rated items on a scale of 0 to 3, which assess a subject's normal "chance of dozing or falling asleep" in everyday scenarios. Values above 10 are regarded as excessive sleepiness during the daytime, and values above 15 as severe daytime sleepiness. The Short-Form Health Survey (SF-36) instrument has 36 items, which are divided into eight categories based on functional capacity, physical limitations, physiological discomfort, overall health perception, vitality, limitations due to social and emotional factors, and mental health. Zero scores represent a worse HRQoL status, while 100 points represent the best HRQoL overview. The Sleep Deprivation Index (SDI) [10] was used to evaluate sleep deprivation by calculating the difference between the mean number of hours slept on weekends and the mean number of hours slept on weekdays. Academic performance was measured by the self-reported grade point average (GPA), ranging from 0 (least) to 4 (highest), based on the percentage marks obtained. To get feedback on the questions’ clarity and readability, the questionnaires were first given to a pilot group of students from various levels.

Statistical analysis

After the survey, the completed forms were coded, compiled, and imported into Microsoft Excel 2019 (Microsoft® Corp., Redmond, WA, USA), and then exported and analyzed using IBM SPSS Statistics for Windows, Version 20 (released 2011; IBM Corp., Armonk, NY, USA). Quality of sleep, daytime sleepiness, sleep deprivation, and HRQoL among dental students were represented as mean ± SD. To test the statistical significance of the association between altered sleep patterns and academic performance, the Pearson correlation test was used. Results with p ≤ 0.05 were considered statistically significant.

## Results

The study involved first- to final-year undergraduate dental students at Amrita University. All students were invited to participate via electronic mail, and participation was voluntary and anonymous. A total of 102 students consented to participate, resulting in a response rate of 57.6%. The mean age of participants was 20 years; among them, 83 were females and 19 were males.

Daytime sleepiness was evaluated using the ESS score, and the mean ESS score was 8.40 ± 4.66. An ESS score of more than 10 indicates excessive daytime sleepiness; 30.3% (n = 31) of students showed daytime somnolence.

For assessing sleep quality, the mean PSQI score was 6.29 ± 2.5, with 63.7% (n = 65) of students showing a global PSQI score of more than 5, indicating poor sleep quality. The mean score of the SDI was 2.58 ± 2.38. The self-rated GPA, used to assess academic performance, had a mean score of 2.89 ± 0.67.

HRQoL was evaluated using the SF-36 under eight different categories. The mean values were as follows: HRQoL physical functioning, 76.86 ± 22.56; physical health, 59.66 ± 38.65; emotional problems, 52.61 ± 39.98; energy/fatigue, 47.75 ± 16.49; emotional well-being, 52.16 ± 17.03; social functioning, 63.81 ± 24.27; pain, 71.56 ± 21.88; and general health, 64.71 ± 10.37. The domains of energy/fatigue, emotional well-being, and emotional problems presented lower scores (Table 1).

**Table 1:** Descriptive statistics of sleep quality, health related quality of life, excessive daytime sleepiness, and sleep deprivation. N, Sample Size; SD, Standard Deviation; PSQI, Pittsburgh Sleep Quality Index; ESS, Epworth Sleepiness Scale; SF-36, 36-Item Short Form Survey

Questionnaires	N	Mean	SD
C1 - Subjective sleep quality	102	1.24	0.76
C2 - Sleep latency	102	0.96	0.90
C3 - Sleep duration	102	1.61	0.86
C4 - Sleep efficiency	102	0.50	0.80
C5 - Sleep disturbance	102	1.15	0.72
C6 - Use of sleep medication	102	0.13	0.53
C7 - Daytime dysfunction	102	0.72	0.82
Global PSQI	102	6.29	2.50
SF-36			
Physical functioning	102	76.86	22.56
Role limitation: physical health	102	59.66	38.65
Role limitation: emotional problem	102	52.61	39.92
Energy/fatigue	102	47.75	16.49
Emotional well-being	102	52.16	17.02
Social functioning	102	63.84	24.24
Pain	102	71.42	21.77
General health	102	64.71	10.37
ESS	102	8.40	4.66
Sleep deprivation index	102	2.58	2.38

**Figure 1:**
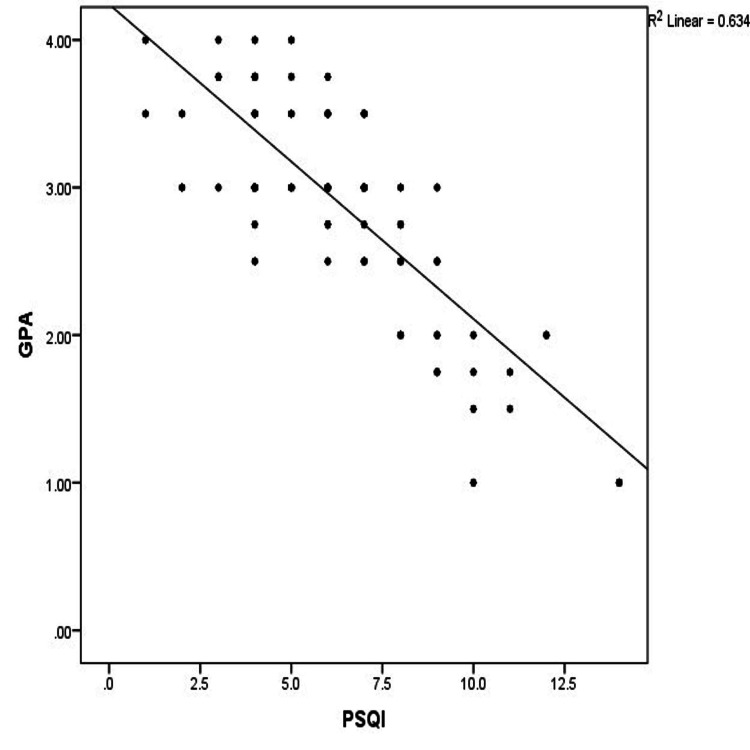
Correlation between academic performance (GPA) and sleep quality (PSQI). PSQI, Pittsburgh Sleep Quality Index; GPA, Grade Point Average

The relationship between quality of sleep (PSQI) and academic performance (GPA) showed a strong negative correlation between PSQI and GPA (Pearson correlation = -0.796, R² = 0.634, p = 0.00). That is, poor sleep quality (PSQI > 5) was correlated with lower academic performance among students, and this finding was statistically significant (Table 2 and Figure 1). Whereas, there was no statistically significant association between excessive daytime sleepiness and academic performance (GPA).

**Table 2:** Correlation of academic performance with sleep quality and excessive daytime sleepiness. *p-value of less than 0.05 was considered statistically significant. N, Sample Size; PSQI, Pittsburgh Sleep Quality Index; ESS, Epworth Sleepiness Scale; GPA, Grade Point Average

Variables	N	GPA Pearson correlation coefficient (r)	p-value
PSQI	102	-0.796	0.00*
ESS	102	0.006	0.95

## Discussion

The present study aimed to assess the influence of altered sleep patterns on HRQoL and academic performance among undergraduate dental students in Kerala. This study found that 63.7% (n = 65) of undergraduate dental students had poor sleep quality, which was similar to earlier research reporting a prevalence of poor sleep quality ranging from 65.2% to 72.5% [1,11]. However, these prevalence rates among dental students are higher compared to the global prevalence rate of 40% among medical students [12]. Furthermore, evidence has shown that dental and medical students are more affected than students pursuing other careers [2]. This may be due to academic workload, long clinical hours, and lifestyle factors that contribute to poor sleep quality [13].

Moreover, the study showed that only 37.3% (n = 38) of undergraduate dental students slept for more than six hours a day, which is the expected normal sleep duration. The average sleeping hours during weekdays were found to be six hours, and during weekends, nine hours. These results were comparable to the average sleep hours reported by previous studies [14,15]. A disturbed sleep cycle mainly leads to daytime somnolence. The study revealed that 30.3% (n = 31) of undergraduate dental students reported excessive daytime sleepiness, which can lead to decreased quality of life, poor work efficiency, and reduced academic performance [16].

Upon assessing the variables of HRQoL examined in this study, it was found that the energy/fatigue domain had the lowest scores. Similar results were shown by Caldeira et al. [7]. Low scores in this domain may be caused by a variety of circumstances, including the development of conceptual, laboratory, and practical procedures; interactions between students, professors, and patients; and the demanding study schedule. The next domain was mental or emotional well-being, which also showed a low score. The transition from adolescence to adulthood, along with challenges such as social adjustments, heavier workloads, increased emotional strain, and the need for technical expertise, has led to a greater mental health burden for students pursuing biomedical science courses [11]. Due to higher stress levels, dental students are more likely to experience psychological problems like anxiety and depression. While data from cross-sectional research [17,18] indicated that the prevalence of anxiety ranged from 31% to 66%, a recent systematic review by Muniz et al. [19] revealed that depression prevalence among dental students was 29% worldwide. Thus, there is a necessity for targeted therapies and support networks in dental education to lessen the mental health difficulties that students encounter, thereby guaranteeing their health and preparedness as future medical professionals [20]. Other domains assessing HRQoL, such as physical functioning, social functioning, pain, and general health, had comparatively better scores among dental students.

The association between academic performance and quality of sleep, daytime somnolence, was also evaluated. It was found that poor sleep quality, which was graded using high PSQI scores, had a strong negative correlation with academic performance (GPA scores). Dental education and practice, particularly during the clinical years, require a high degree of focus, mental agility, and dexterity. Lack of sleep, or poor-quality sleep, can negatively impact performance [21]. Results from previous literature, such as Muñoz et al., El Hangouche et al., and Okano et al. [1,22,23], also support the finding that poor sleep quality and shorter sleep duration lead to lower academic performance. Poor sleep quality can cause fatigue throughout the day, thereby impacting students' academic performance. A recent systematic review among university students suggests that insufficient sleep has adverse effects on academic achievement. Similarly, a meta-analysis of 15 studies with 10,240 samples showed positive correlation coefficients, indicating a link between better sleep quality and improved academic performance [12]. This study’s results did not show any association between excessive daytime sleepiness and academic performance, even though 30.3% (n = 31) of the students reported excessive daytime sleepiness. This finding was supported by El Hangouche et al. and Al-Zahrani et al. [22,24]. Nonetheless, other earlier research supports the idea that students' academic performance and motivation can be affected by daytime sleepiness [4]. Excessive daytime sleepiness may be caused by a variety of factors. University students with shorter nocturnal sleep duration or inconsistent sleep-wake schedules are more likely to experience daytime tiredness. Daytime fatigue was more common in students with shorter nighttime sleep durations or irregular sleep-wake patterns [25].

Keeping a regular sleep schedule is essential to preserving both physical health and emotional well-being. Research suggests that optimal neurocognitive and psychomotor function can only be attained with a healthy sleep cycle [26]. The results and findings of this study demonstrate and highlight the significance of having good sleep quality, leading to improved HRQoL and academic performance among dental students.

One of the key limitations of the study was the reliance on self-reported GPA, instead of obtaining official academic records from the university registrar, which may affect the accuracy of the academic performance data. Additionally, the proportion of male participants was notably lower than that of females, which is a common trend across Indian dental schools and may potentially limit the generalizability of the findings.

Furthermore, several limitations are either implicit or not fully addressed. These include the cross-sectional design, which restricts the ability to draw causal inferences; reliance on self-reported data, which may introduce recall and reporting biases; lack of control for potential confounding variables, such as stress levels, socioeconomic status, or lifestyle habits; and limited generalizability due to the single-institution sample and gender imbalance.

To address these limitations, future research should aim to obtain official academic transcripts or secure access to university-recorded GPAs to ensure the accuracy and reliability of academic performance data. Where direct access is not feasible, alternative objective indicators - such as standardized test scores or course-specific grades - could be used in conjunction with self-reported data. Furthermore, larger multicenter and longitudinal studies are recommended to better understand the underlying causes of sleep disturbances and to inform the development of effective preventive strategies aimed at improving academic outcomes in university students. Incorporating qualitative approaches in future research could also provide a more comprehensive understanding of sleep health in dental students by capturing contextual and experiential factors.

## Conclusions

The study findings show that dental students frequently experience inadequate or poor sleep quality, daytime somnolence, and psychological, emotional, and mental distress. There have been strong correlations shown between poor sleep quality and subpar academic achievement. This justifies focusing instructional interventions on this crucial facet of dental students’ lives. These findings allow educators and students to better understand the negative consequences of sleep disruptions on academic performance. Therefore, it is strongly advised that dental students take necessary steps to enhance their sleeping patterns, improving their academic, overall health, and psychological well-being.
